# Comparison of efficacy according to voltage of pulsed radiofrequency treatment to lumbar dorsal root ganglion in patient with lumbar radiculopathy: Pilot study

**DOI:** 10.1097/MD.0000000000033617

**Published:** 2023-04-28

**Authors:** Jae Ni Jang, Soyoon Park, Ji-Hoon Park, Yumin Song, Sooil Choi, Young Uk Kim, Sukhee Park

**Affiliations:** a Department of Anesthesiology and Pain Medicine, International St. Mary’s Hospital, Catholic Kwandong University School of Medicine, Incheon, Korea; b Department of Anesthesiology and Pain Medicine, Dongsan Medical Center, Keimyung University School of Medicine, Daegu, Korea.

## Abstract

**Background:**

Lumbar radicular pain (LRP) is a common symptom, but a challenging clinical problem. Pulsed radiofrequency (PRF) is a more recently developed technique that uses short pulses of radiofrequency current with intervals of longer pauses to prevent temperature from rising to the level of permanent tissue damage and has been advocated in treatment of such patients. But there were no comparative studies on the analgesic effects according to output voltage during PRF in patients with LRP. The goal of this study is to determine the clinical effect of high-voltage (60V) versus standard-voltage (45V) PRF of lumbar dorsal root ganglion.

**Methods/design:**

This study will be a prospective, double-blind randomized controlled pilot study. In this study, total 20 patients will be recruited and distributed equally into 2 groups: high-voltage (60V) PRF, low-voltage (45V) PRF. Outcomes will be radicular pain intensity; physical functioning; global improvement and satisfaction with treatment; and adverse events. The assessments will be performed at the 3-month follow-up period after the end of the treatments. The findings will be analyzed statistically considering a 5% significance level (*P* ≤ .05).

**Discussion:**

The results of this trial will help determine which voltage could be applied for PRF to dorsal root ganglion in LRP and be a basis for subsequent trials.

## 1. Introduction

Lumbar radicular pain (LRP) is a common symptom, but a challenging clinical problem that involves radiating pain in one or more dermatomes and may be accompanied by chronic nerve irritation and dysfunction.^[1,2]^ Interventional procedures may be considered if conservative treatments, such as physical therapy or medication are initially tried and are not effective.^[2]^

Pulsed radiofrequency (PRF) of interventional procedures is a more recently developed technique that uses short pulses of radiofrequency current with intervals of longer pauses to prevent temperature from rising to the level of permanent tissue damage.^[3,4]^ Although the mechanism of PRF is still not fully understood, PRF is easily accepted as a nerve modulation rather than nerve destruction. Previous studies have shown that this treatment is safe and effective in treating various painful disorders including LRP by intermittently applying high-frequency currents adjacent to the lumbar dorsal root ganglion (DRG).^[5,6]^ However, there have been controversy results about the PRF effect of lumbar DRG.^[7]^ In addition, the effect applied to lumbar DRG did not seem to last longer than PRF applied to other areas.^[8]^

So, subsequent studies should examine whether increasing the dosage and modifying intraoperative parameters of PRF can improve its effects. Luo et al^[9]^ suggested that analgesic efficacy was positively correlated with output voltages during PRF in trigeminal neuralgia. To our knowledge, there has been no comparative study on the analgesic effects according to output voltage during PRF in patients with LRP. Thus, in this study, we will aim to assess the clinical effects of high-voltage (60V) versus low-voltage (45V) PRF of the lumbar DRG.

## 2. Materials and methods

### 2.1. Trial registration

This trial has received complete ethical approval from the Ethics Committee of Catholic Kwandong University International St. Mary’s Hospital (IS22OISE0032). This trial was registered at the Clinical Trial Registry of Korea (CRIS, www.cris.nih.go.kr.) (Registration number KCT0007578).

### 2.2. Trial design

**Figure 1.**
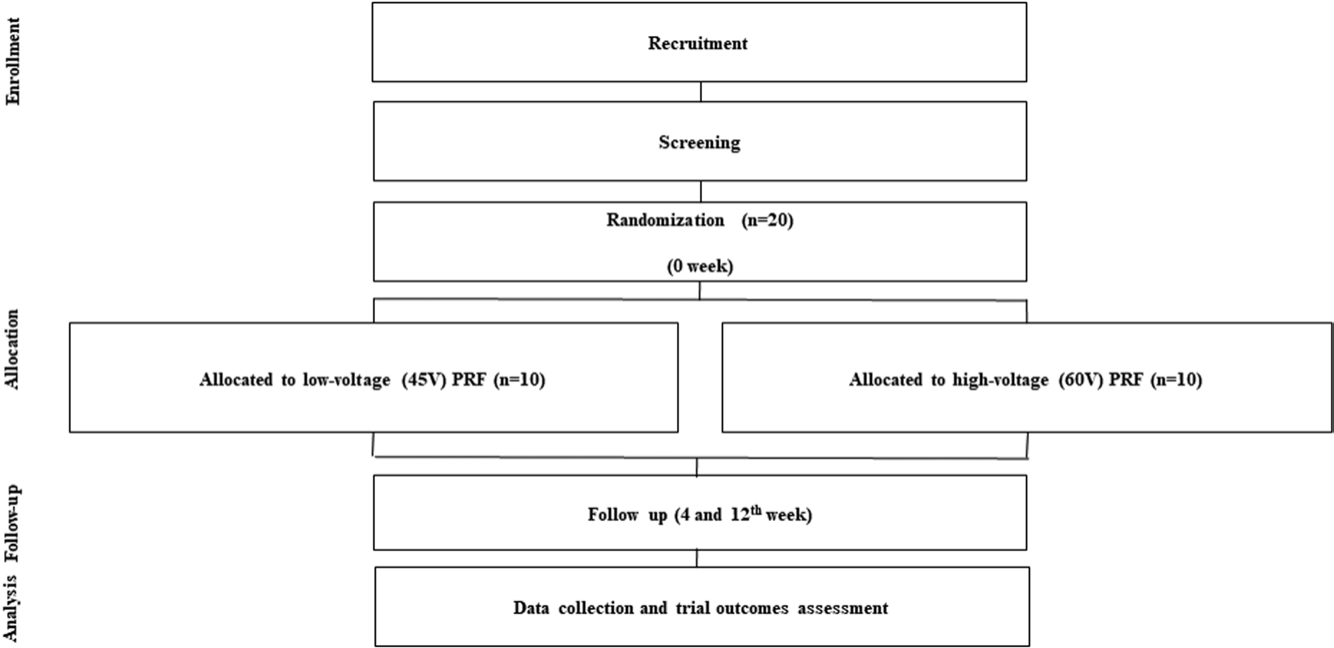
Flowchart of study design. PRF = pulsed radiofrequency treatment.

As a part of the ethical approval process, we will provide a written informed consent form to the participants before their enrollment in the study. This study will be a double blind, randomized controlled trial to compare the 2 groups (1:1). This study will be performed in 20 participants with LRP and conducted on outpatients visiting the pain clinic of International St. Mary’s Hospital, Catholic Kwandong University. The clinical trial and the items to be examined in this study are presented in Figure 1.

### 2.3. Inclusion criteria

1. Age ≥20 years old
2. Pain intensity ≥5 out of 11 on the numerical rating scale (NRS)
3. Chronic LRP lasting ≥12 weeks
4. Previous failure of conservative management such as physiotherapy, exercise therapy, or analgesic medications
5. Lumbar spinal stenosis confirmed by magnetic resonance imaging
6. Patients who received conventional fluoroscopy-guided diagnostic/therapeutic transforaminal epidural injections with local anesthetics and steroids
7. Patients who reported persistent pain (NRS score ≥5) after receiving TFESI
8. Patients who received at least 2 epidural steroid injections in the 3 months preceding PRF treatment

### 2.4. Exclusion criteria

1. Patient refusal
2. Age <20 years
3. Unbearable pain >9–points on NRS, pain <4–points on NRS
4. Acute pain of onset lasting <12 weeks
5. Signs of progressive motor weakness or neurologic deficits
6. Allergies to steroids or contrast dyes
7. Coagulopathy
8. Epidural steroid injection within the previous 4 weeks
9. Systemic infection, injection site infection
10. Malignancy

### 2.5. Assignment of interventions and blinding

Participants will be randomized using a computer-generated scheme to either treatment group: low-voltage group and high-voltage group. Immediately prior to the procedure, procedure-performing physician (S.P.) will open a sequentially numbered opaque envelope with group assignment listed inside. All participants will remain blind to the group assignment before, during, and after the PRF and all efforts will be made to provide identical treatment experiences. The injection procedure and type of drug used for treatment will be not revealed to the patients until study completion. The researcher who gathered post-procedure data also will be blind to group assignment.

### 2.6. Procedures

All procedure will be performed under fluoroscopic guidance. A single fluoroscopy C-arm system will be used. The patient will be placed in the prone position with a pillow under the lower abdomen. After sterile preparation of the needle insertion area, the skin will be infiltrated with 1% lidocaine, and a 22-guage, 4-in RF cannula with a 10 mm curved active tip will be advanced under fluoroscopic guidance. After appropriate positioning of the RF cannula, the stylet will be replaced by the RF probe and the probe will be connected to the PRF generator (Radiofrequency Ablation for Pain Management, G4™ RF Generator; Cosman Medical, Burlington, NJ). The final definite position of the RF probe will require a sensory stimulation (50 Hz) threshold ≤0.4 V, which create paresthesia corresponding to the existing distribution of the patient’s radicular pain. The motor stimulation threshold (2 Hz) should be more than 1.5 times greater than the sensory stimulation threshold, and impedance <400 Ω is also required. The position of the RF cannula will be slightly adjusted after each cycle of treatment, and the motor/sensory stimulation will be also performed at the same time for confirmation.

In experimental high-voltage group, the output voltage will be set to 60V with temperature no more than 42°C, pulsed width of 20 millisecond, frequency at 2 Hz, and duration for 120 seconds and 3 cycles of PRF will be applied. In low-voltage group, only the output voltage will be different to 45V compared to experimental group.

The generator will be manipulated by the operating room nurse, and the display will be concealed from the patient and the procedure-performing physician. After PRF, the needle will be further retreated and then repositioned at the safe triangle. After confirmation of epidural spread using a contrast dye, 1 mL of 0.3% mepivacaine will be injected.

### 2.7. Outcome assessment and follow-up

As part of the baseline data, we will include information such as age, sex, height, weight, body mass index, coexisting medical conditions (e.g., diabetes and hypertension), diagnosis, total duration of pain, target level of the affected nerve root, and the number of prior epidural injections. For intra-operative information, we will collect the stimulating voltages used during electrical stimulation positioning with 50 and 2 Hz of electrical stimulation, procedure duration, output voltage, impedance, and electric field intensity ([output voltage]2/resistance).

Outcome measures will include: radicular pain intensity; physical functioning; global improvement and satisfaction with treatment; and adverse events. An 11-point NRS (0 = no pain, 10 = unbearable pain) will be used to assess radicular pain intensity; the Korean version 10-item Oswestry disability index (ODI) questionnaire (range: 0–100; 0 = no disablility) will be used to assess physical function; global perceived effect according to the 7-point Likert scale will be used to assess patient satisfaction and improvement; and adverse events during treatment and follow-up will be individually recorded.

The primary outcome will be NRS and ODI at 1, 2, and 3 months after the procedure. The reduction in pain intensity and decrease in ODI compared with baseline. Complications that occur during the procedure will be reported, if present, and adverse events will be further evaluated during the monthly follow-up visits.

### 2.8. Statistical analysis

The intention-to-treat analysis will be applied, and the data of every randomized subject will be analyzed each month, regardless of lost to follow-up or withdrawal from the study. Follow-up loss or withdrawal from the study will be considered treatment failure. Because we expect possible treatment failure during the 3-month follow-up period, linear mixed model will be used to analyze the secondary continuous variables. Adjustments will be made for the baseline values to 0 to compare the decrements of the secondary outcomes at 3 months. The categorical variables will be presented as absolute numbers and percentage. The continuous variables will be presented as the mean with standard deviation, 95% confidence interval, or median and interquartile range. To compare the demographic data of the 2 groups, the chi-square or Fischer exact tests will be used to assess categorical variables and the unpaired *t* test or Mann–Whitney *U* test will be used to analyze continuous variables. *P* values <.05 will be considered statistically significant. All statistical analyses will be performed using SPSS 26.0 (IBM Corp., Chicago, IL).

## Author contributions

**Conceptualization:** Young Uk Kim, Sukhee Park.

**Data curation:** Jae Ni Jang, Soyoon Park, Sukhee Park.

**Formal analysis:** Soyoon Park.

**Funding acquisition:** Sukhee Park.

**Investigation:** Jae Ni Jang, Ji-Hoon Park, Yumin Song, Sukhee Park.

**Methodology:** Jae Ni Jang, Ji-Hoon Park, Sooil Choi, Young Uk Kim, Sukhee Park.

**Resources:** Yumin Song.

**Supervision:** Sukhee Park.
